# Five-year Trends in Opioid Prescribing Following Orthopaedic Trauma

**DOI:** 10.5435/JAAOSGlobal-D-20-00134

**Published:** 2020-08-20

**Authors:** Christopher D. Flanagan, Noah M. Joseph, Alex Benedick, Heather A. Vallier

**Affiliations:** From the Department of Orthopaedic Surgery, MetroHealth Medical Center, Cleveland, OH, affiliated with Case Western Reserve University.

## Abstract

**Introduction:**

Legislatures across the country are passing new opioid prescribing laws. To understand the effects of this legislation, baseline autonomous shifts in physician opioid prescribing must be evaluated.

**Methods:**

The purpose of this retrospective dual cohort comparison study was to evaluate 5-year opioid prescribing trends in orthopaedic trauma patients. Demographic and injury information were collected on adult trauma patients with surgically managed orthopaedic fractures from 2012 (N = 190) and 2017 (N = 160). The amount of opioid medication prescribed from discharge to 1 year after the injury was collected. Opioid prescriptions were converted to morphine milligram equivalents (MMEs). The main outcome measure was opioid medication prescribed in 2017 versus 2012.

**Results:**

The cohorts were well-matched on sex, race, medical comorbidities, substance use, fracture location, Injury Severity Score, hospital length of stay, and intensive care unit admission metrics. However, the 2012 cohort was older than the 2017 cohort (51.9 versus 43.3 years, *P* < 0.001). When controlling for age, total opioid medication prescribed was greater in 2012 than in 2017 (1,680 versus 1,110 MME, *P* = 0.001). Patients in 2017 received both lower discharge prescriptions (523 versus 407 MME, *P* < 0.001) and lower total opioid prescription refill amounts (1,140 versus 766 MME, *P* = 0.037). The number of refills prescribed was equal, but patients received lower amounts of opioid medications per refill in 2017 (333 versus 243 MME, *P* < 0.001). Despite these differences, the percentage of patients ceasing prescription opioid use 1 year after injury was unchanged (90.6% versus 92.1%, *P* = 0.675).

**Discussion:**

Over 5 years, providers have successfully reduced the amount of opioid medication prescribed to surgically managed orthopaedic trauma patients through self-directed measures. The effects of opioid prescribing legislation should be viewed from this baseline.

The opioid crisis in the United States has had devastating consequences, with approximately 130 people dying each day from opioid overdoses.^1^ The economic impact has also been substantial, with the costs of prescription opioid abuse estimated at $78.5 billion in 2013.^2^ The role healthcare providers have played in contributing to this national epidemic has been the subject of much conversation.^3,4^ From these discussions, an apparently simple solution has emerged: prescribe fewer opioid medications. Multiple studies have demonstrated that reducing opioid prescriptions may diminish long-term opioid use.^5,6^ This, in combination with interventions such as increasing regional anesthesia use and providing nonopioid medications for pain control, has been advocated to reduce opioid dependence while providing acceptable analgesia.^7-9^

In certain populations, opioid analgesia is often appropriately indicated to achieve acceptable pain relief. This is the case with orthopaedic trauma patients who present with acute injury. However, given the evidence that judicious opioid prescribing can reduce long-term adverse consequences, it is important to assess whether prescribers are modifying their practice patterns. Therefore, the purpose of this study was to compare opioid prescribing during 2012 with that of 2017 for surgically managed orthopaedic trauma patients. Specifically, the goal was to assess total postinjury opioid prescribing, as well as the individual components of initial discharge and refill prescriptions.

On August 31, 2017, the legislature of the study state issued a new a law, effective January 1, 2018, that limited the number of days for which opioids could be prescribed. Certain aspects of this law exempted orthopaedic surgeons; specifically, surgeons could continue to prescribe opioids per their discretion if a reason for the prescription was documented. Therefore, the purpose of this study was to assess how opioid prescribing practices for surgically managed orthopaedic trauma patients had evolved immediately before the implementation of these new regulations. This information would help identify both autonomous changes in orthopaedic provider prescribing habits and serve as a baseline to measure the effects of the new legislation. We postulated that orthopaedic prescribers would autonomously respond to concerns related to opioid prescribing and take steps to reduce their opioid footprint, even before legislative mandate.

## Methods

### Design and Setting

This was a retrospective dual cohort study evaluating orthopaedic trauma patients from 2 calendar years who presented to a single level 1 trauma center in a major metropolitan area of the Midwest region of the United States. Institutional Review Board approval was obtained for the study.

### Selection Criteria

Adult patients who presented for evaluation of traumatic injury and met the following eligibility criteria were included in this study: (1) sustained fractures of the upper extremity, lower extremity, pelvis, and/or spine; (2) underwent fixation of at least one fracture; (3) admitted for at least one midnight; and (4) minimum of one postoperative follow-up visit. Patients with chronic opioid use at the time of presentation were excluded from the study. Patients from 2 calendar years who met these criteria were included: 2012 (N = 190), 2017 (N = 160).

### Data Collection

Demographic information including age at injury, sex, race, number of medical comorbidities, and the use of alcohol or tobacco before admission were recorded. Hospital stay characteristics were also summarized, including fracture location, Injury Severity Score, length of stay, and the presence of an ICU admission.

All opioid medications prescribed to patients at the time of discharge and for 1 year after injury were recorded. Opioid medication was defined using FDA classifications; the opioids included in this analysis were morphine, oxycodone, hydromorphone, fentanyl, oxymorphone, codeine, methadone, and tramadol. Although codeine and tramadol are regulated differently from the other medications, the FDA defines both as opioid pain relievers, so each was included in this analysis.^10,11^

Most patients were admitted to either the orthopaedic or trauma surgery service. At the study institution, most opioid prescriptions are written by rotating residents. Within the orthopaedic department, there are greater than 20 different rotating residents annually who are responsible for opioid prescribing. This is in addition to at least an equivalent number of rotating residents on the trauma surgery service. Therefore, these data capture a large volume of unique prescribers.

Two systems were used for evaluating opioid prescribing. In 2012, no mandatory, state-reporting system for controlled substances was in place. Therefore, all opioid use data were generated from chart review within the electronic medical records. Starting in 2017, the state began using a mandatory reporting system for controlled substances. To maintain parity, the opioid discharge prescription was obtained from the EMR. However, for the 2017 patient cohort, the mandatory state-reporting system was used to quantitate opioid prescribing. Opioid prescriptions in both groups were collected for 1 year after injury. During the study periods, there were no institutional or state policies in place, mandating opioid prescribing practices; the amount of opioid prescribed was based on the individual provider's clinical judgement. All opioid prescriptions were converted to morphine milligram equivalents (MMEs) using a conversion chart based on standard equianalgesic opioid data.^12^ As a reference, 10 mg oxycodone represents 15 MME.

Several aspects of opioid prescribing were quantified. Total opioid medication prescribed represented the sum of the discharge prescription and all refills. The discharge prescription MME and total refill MME data were also collected and reported separately. In addition to the total refill MME, the number of refills prescribed was also recorded, as was the mean amount prescribed per refill. Only patients who received a refill were included in this derivation. Given that prescribing habits may have shifted toward weaker narcotics, data were also analyzed excluding tramadol use. Finally, the number of patients off of prescription opioids at 1 year after injury was collected. Prescription opioid cessation was defined as 3 months without a prescription for opioids.

### Analysis

Univariate analysis was completed with Prism 8.0a software (GraphPad Software Inc). Chi-square, Fisher exact, and Student *t*-tests, with Welch corrections in the setting of unequal variance, were generated in the appropriate setting. Because previous research has described a negative correlation between age and opioid use,^5,13,14^ age-matched cohorts were derived from the larger groups for more equitable comparison. To age-match the groups, each cohort was subdivided into 10-year age categories (<21, 21 to 30, …, 61 to 70, 71+), and the youngest and oldest age groups were excluded. All statistical analyses were two-tailed. Statistical significance was set to *P* < 0.05 for all outputs.

## Results

### Patient Demographic and Injury Characteristics

The two groups were well-matched on both demographic and injury characteristics. Demographic characteristics in each group parodied reported national statistics for level 1 trauma patient populations.^15^ Trauma patients in both groups were predominantly male (65.8% versus 66.9%) and Caucasian (72.1% versus 65.6%). Tobacco and alcohol use were moderately prevalent and equivalent in both groups (alcohol: 41.6% versus 34.4%, *P* = 0.19; tobacco: 35.3% versus 26.9%, *P* = 0.11). There was no difference in the mean number of associated medical comorbidities between groups, and the cohorts had a comparable degree of injury, as defined by the location of fracture and Injury Severity Score (both 11). Hospital stay characteristics were also analogous based on the percentage of patients admitted to the ICU well as average length of stay. However, the 2012 cohort was older than the 2017 cohort (51.9 versus 43.3 years, *P* < 0.001) (Table 1). Excluding the youngest and oldest patients in each group produced appropriately age-matched subcohorts (2012: N = 138; 2017: N = 140) (44.4 versus 43.2 years).

**Table 1:** Injury and Hospital Stay Data Are Presented for Patients Treated in 2012 Versus 2017

	2012 (N = 190)	2017 (N = 160)	*P* Value
Sex			
Male	125 (65.8%)	107 (66.9%)	0.91
Female	65 (34.2%)	53 (33.1%)	
Race			
Caucasian	137 (72.1%)	105 (65.6%)	0.23
Black or African American	45 (23.7%)	50 (31.3%)	
Other or unknown	9 (4.7%)	5 (3.1%)	
Age (yr)	51.9	43.3	<0.001
Substance use			
Alcohol	79 (41.6%)	55 (34.4%)	0.19
Tobacco	67 (35.3%)	43 (26.9%)	0.11
Comorbidities	1.9	1.6	0.25
ISS	11.1	11.4	0.64
Orthopaedic injury			
Upper extremity fracture	54 (28.4%)	41 (25.6%)	0.47
Lower extremity fracture	134 (70.5%)	113 (70.6%)	
Pelvic fracture	23 (12.1%)	25 (15.6%)	
Spine fracture	20 (10.5%)	25 (15.6%)	
Length of stay (d)	6.2	5.6	0.35
ICU admission	45 (23.7%)	31 (19.4%)	0.36

ISS = Injury Severity Score

Data are presented as total numbers (and percent) or as mean age, number of comorbidities, ISS, and length of stay.

### Opioid Prescribing Trends

Oxycodone was the major opioid prescribed in both groups. In 2012, oxycodone accounted for 81.2% of all prescription MME; for 2017, this amount was 89.6% of all prescription MME. When the full cohorts were analyzed, no differences were seen between groups in total opioid medication prescribed from discharge to 1 year follow-up (1,430 versus 1,170 MME, *P* = 0.122) (Table 2). Dividing total prescriptions into discharge prescription and refill subcategories, patients in 2017 were prescribed less opioid medication at discharge than patients in 2012 (499 versus 414 OME, *P* = 0.008). However, total opioid refill amounts were equal between groups (881 versus 790 MME), even when excluding tramadol prescriptions (854 versus 741 MME), with both groups receiving the same number of refills (2.8 versus 3.2). However, patients did receive a lower amount of opioid medication per refill in 2017 than in 2012 (332 versus 249 MME, *P* < 0.001). An equivalent percentage of patients were off of opioid medications within 1 year of injury (over 90%).

**Table 2:** Mean Opioid Prescribing Trends Are Shown for All Patients Treated in 2012 Versus 2017 and for the Age-Matched Cohorts From Each Period

	Overall			Age-Matched		
	2012 (N = 190)	2017 (N = 160)	*P* Value	2012 (N = 138)	2017 (N = 140)	*P*-Value
Total opioid medication prescribed (MME)	1,430	1,170	0.12	1,680	1,110	0.001
Discharge prescription (MME)	499	414	0.008	523	407	<0.001
Refill opioid use (MME)	881	790	0.53	1,140	766	0.037
Refill opioid use (excluding tramadol) (MME)	854	741	0.49	1,050	702	0.038
No. of refills	2.8	3.2	0.52	3.6	3.4	0.81
Opioid amount per refill (MME)	332	249	<0.001	333	243	<0.001
Off opioids at 1 yr	177 (93.2%)	149 (93.1%)	1.00	125 (90.6%)	129 (92.1%)	0.68

MME = morphine milligram equivalent

Controlling for age, total opioid medication prescribed was greater in 2012 than in 2017 (1,680 versus 1,110 MME, *P* = 0.001). Differences in discharge prescription amount and total refill opioid use were also apparent in this subgroup analysis, with patients in 2017 receiving both lower opioid discharge prescriptions (523 versus 407 MME, *P* < 0.001) and lower total opioid refill amounts (1140 versus 766 MME, *P* = 0.037). The reduction in discharge prescription amount is equivalent to reducing prescriptions from approximately 70 oxycodone 5 mg tablets in 2012 to 55 oxycodone 5 mg tablets in 2017. These results continued to be true when excluding tramadol prescriptions (1,050 versus 702 MME, *P* = 0.038). The number of refills prescribed was equal between groups (3.6 versus 3.4), and patients received lower numbers of opioid medications per refill in 2017 than in 2012 (333 versus 243 MME, *P* < 0.001). Despite these differences, the percentage of patients who had ceased using prescription opioids 1 year after injury was unchanged (Table 2).

## Discussion

The current opioid crisis is one of the foremost public health concerns in the United States. As the complex factors at the heart of the epidemic come to light, the healthcare system has recognized its role in promoting, and now reversing, the widespread use of opioid pain medication. Although some patients, such as those with major orthopaedic injury, may have reasonable indications for opioid use, prescribers must recognize the goal of providing relief with the most conservative dose possible for the shortest period of time. Therefore, the purpose of this study was to identify trends in opioid medication prescribing to orthopaedic trauma patients with surgically managed fractures during 2012 versus 2017.

When controlling for age, orthopaedic trauma patients with surgically managed fractures in 2017 received less opioid medication at all times compared with patients in 2012. The total amount of opioid medication prescribed within in 1 year after injury, the opioid discharge prescription, the total refill opioid amount, and the opioid amount prescribed per refill were all lower in 2017 than in 2012. This was because of reduced prescribing overall and not exclusively to a transition to prescribing weaker opioids, such as tramadol or codeine. Despite these changes, an equivalent percentage of patients had ceased prescription opioid use within 1 year after injury. These results suggest that prescribers are more aware of their contribution to the current crisis and have taken appropriate steps to reduce their opioid footprint. These modifications were self-directed and not taken in response to new state or institutional prescribing regulations.

There are several limitations to this study. First, no mandatory, state-reporting system for controlled substances was in place in 2012. Therefore, for this cohort, only opioid prescriptions originating within the single study institution were tabulated. Therefore, for this cohort, only opioid prescriptions originating within the study institution were tabulated, which could lead to under-reporting in the 2012 cohort. Because notable differences are reported in total opioid use, discharge prescription, refill amount per prescription, and refill opioid use despite this possible under-reporting, these results remain valid despite the 2012 limitation. However, it is possible that the number of refills and the number of patients off of opioids at 1 year are not fully captured given the 2012 limitation. Second, this study does not attempt to assess patient satisfaction with pain control between the 2 years. It is possible that because of the decrease in opioid pain medication prescribed, patients in 2017 felt their pain was less adequately controlled than patients in 2012. However, both an increased number of refills and a longer period of opioid prescribing might be expected in the setting of inadequate pain control. This did not occur in our study. The same number of refills were provided for each of the groups, and no increase in the percentage of patients using opioids 1 year postinjury was seen. This suggest, circumstantially, no difference in overall pain control. Finally, this study does not attempt to correlate reductions in opioid prescribing with changes in nonprescription opioid use, opioid dependence, or opioid addiction. More research is necessary to determine the societal effects of reduced opioid prescribing.

Physician autonomy is necessary to provide optimal patient care. However, when physicians fail to appropriately self-regulate, nonphysician oversight bodies will mandate change, with or without physician input. This study suggests that orthopaedic prescribers have responded appropriately to concerns regarding the correlation between opioid prescribing and pathologic opioid use and dependence. Through self-modification of prescribing practices, without binding legislative, regional, or institutional regulations, providers have reduced the amount of opioid medication available to orthopaedic trauma patients, without a concomitant increase in refill the numbers or sustained opioid use. More research is necessary to determine the effects of these modifications on patient satisfaction and nonprescription opioid use trends.
